# Seroprevalence and risk factors for COVID-19 in the metropolis of the Brazilian Amazon

**DOI:** 10.1038/s41598-022-12629-z

**Published:** 2022-05-20

**Authors:** Maria Karoliny da Silva Torres, Felipe Teixeira Lopes, Aline Cecy Rocha de Lima, Carlos Neandro Cordeiro Lima, Wandrey Roberto dos Santos Brito, Bernardo Cintra dos Santos, Renata Santos de Sousa, Jayanne Lilian Carvalho Gomes, Bruno José Sarmento Botelho, Ana Carolina Alves Correa, Luiz Fernando A. Machado, Rosimar Neris Martins Feitosa, Sandra Souza Lima, Izaura Maria Vieira Cayres Vallinoto, Antonio Carlos R. Vallinoto

**Affiliations:** 1 Laboratory of Virology, Institute of Biological Sciences, Federal University of Pará (Universidade Federal Do Pará-UFPA), Belém, Pará, Brazil; 2 Graduate Program in Biology of Infectious and Parasitic Agents, Belém, Brazil

**Keywords:** Immunology, Viral infection, Epidemiology, Population screening

## Abstract

The devastating effect of health system overload was observed after cases of severe acute respiratory syndrome coronavirus 2 (SARS-CoV-2) peaked in April 2020 in Belém, a capital metropolis in the Brazilian Amazon. Our results showed a high seroprevalence (39.24%) of anti-SARS-CoV-2 IgG antibodies among the population of the capital of the state of Pará after the first wave of the pandemic. Old age, mixed race, a high school education level, low income and contact with infected individuals are risk factors and may be driving seropositivity and exposure to SARS-CoV-2 in this population. This study is one of the first to provide important information to understand the socioeconomic inequalities, behavioral characteristics and viral transmission dynamics associated with the risk of SARS-CoV-2 infection in the capital of the state of Pará, northern Brazil.

## Introduction

On December 8, 2019, in the city of Wuhan, Hubei Province, China, the occurrence of cases of an unusual respiratory syndrome (coronavirus disease 2019—COVID-19) associated with a novel Betacoronavirus (severe acute respiratory syndrome coronavirus 2—SARS-CoV-2) was reported, and a pandemic was decreed on March 11, 2020^1^. Thus, China was considered the initial epicenter of the spread of SARS-CoV-2.

Outside the Asian continent, two new epicenters became prominent: Italy, with the first case identified at the end of February 2020 and cases peaking between the end of March and mid-April 2020^2^, and the United States, where cases first peaked around April 2020; currently, the USA is the most affected country in the world^3^.

With the rapid spread of the virus on the American continent, Brazil became the leading country in terms of the number of SARS-CoV-2 infections in South America. The first report of SARS-CoV-2 infection in Brazil occurred on February 25, 2020, and as of August 18, 2021, the country had amassed 20,416,183 confirmed cases and was the third most affected country in the world^4^. The state of Pará, northern Brazil, has recorded 578,383 confirmed cases and approximately 16,280 deaths^4^. In the city of Belém, the capital of the state of Pará, 105,507 cases were confirmed, and 5077 deaths had occurred by August 17, 2021^5^. The lethality rate in the city of Belém (4.81%) was higher than that in the state of Pará (2.81%)^5^.

To evaluate viral exposure at the population level, the commonly used serological assays aim to identify the presence of antibodies specific to the nucleocapsid (N) or spike (S) protein of SARS-CoV-2, with high sensitivity and specificity^6–9^. Seroprevalence data are essential for understanding the prevalence of subclinical infections, determining the susceptibility of the population to the virus, estimating the actual number of people previously exposed, generating information that provides support for the formulation of public health policies to reduce cases of infection, improving planning for future outbreaks and, finally, evaluating the effectiveness of restrictive measures for containing the spread of the virus.

In this context, the objectives of this study were to describe the seroprevalence of anti-SARS-CoV-2 IgG antibodies and the epidemiological aspects of the risk of exposure to the virus and to determine the proportion of asymptomatic or subclinical infections in residents of the city of Belém, Pará, Brazil, approximately 6 months after the first wave of COVID-19 in the capital city.

## Methods

### Study design and sampling

This was a cross-sectional study conducted with 736 volunteers living in the city of Belém, the capital of Pará, from October 2020 to February 2021, after the first wave of COVID-19. The study was submitted to and approved by the National Research Ethics Committee (CONEP, acronym in Portuguese) and the Human Research Ethics Committee of the Health Sciences Institute of Federal University of Pará (UFPA) under CAAE n. 31,800,720.1.0000.0018 in compliance with the guidelines and regulatory standards for research involving human beings, with all methods conducted in accordance with the Declaration of Helsinki. Individuals already vaccinated with one or two doses against SARS-CoV-2, those who did not complete the epidemiological questionnaire, those who did not sign the informed consent form and those younger than 7 years were excluded from the study.

### Data collection

After obtaining a signed informed consent form for participation in the study, individuals were interviewed using a structured questionnaire addressing clinical, demographic and behavioral characteristics as possible risk factors for SARS-CoV-2. We obtained signed informed consent forms from individuals aged 18 years or older. Children aged 7–11 years and adolescents aged 12–17 years signed a free and informed assent form, and their parents or guardians signed an informed consent form.

The following individual data were collected: (1) sociodemographic data—age, sex, income, ethnicity and marital status; (2) comorbidities; (3) behavioral information on preventive measures, such as mask use, travel, hand hygiene, social distancing and contact with people infected with SARS-COV-2; and (4) information related to COVID-19—the presence of symptoms, previous diagnosis, self-medication and medication prescribed to treat symptoms, hospitalization and the need for mechanical ventilation. The participants’ data were recorded using EPI Info™ software version 7.2.4.^10^ and stored in the local server of the Virology Laboratory of UFPA.

### Sample collection, processing and storage

After questionnaire completion and collection of signed informed consent forms, venous blood samples (10 ml) were collected from the volunteers by venipuncture into a vacuum tube containing EDTA. Subsequently, the samples were processed anonymously in the Virology Laboratory; plasma and leukocytes were isolated and then stored at − 70 °C.

### Serological analysis

Anti-SARS-COV-2 IgG antibodies were detected by ELISA (Euroimmun, Lübeck, Germany), which uses the recombinant structural protein (S1 domain) of the S protein as an antigen, following the manufacturer's protocol. The samples were classified as nonreactive (ratio < 0.8), indeterminate (0.8 ≤ ratio ≤ 1.1) or reactive (ratio > 1.1) for IgG, as suggested by the manufacturer. The manufacturer reported a clinical sensitivity of 75–93.8% (> 10–20 days to ≥ 21 days after disease onset) and a specificity of 99.6% for IgG antibodies.

### Statistical analysis

The seroprevalence estimated based on the sociodemographic characteristics of the population is presented as a count and a percentage with a 95% confidence interval (CI), which were calculated in the program BioEstat 5.0. Correlations between symptoms and positivity were estimated using BioEstat 5.0. Associations between the presence of anti-SARS-CoV-2 IgG antibodies and the study variables were estimated using univariate analysis with the chi-square test or G test. A significance level of 5% (p < 0.05) was established. Uni- and multivariate logistic regression analyses were used to explore associations between risk factors (sociodemographic and behavioral characteristics and symptoms) and SARS-CoV-2 infection and between symptomatic and asymptomatic cases and the presence of antibodies. The definitions established by the Brazilian Ministry of Health were used as a classification criterion for symptomatic individuals^11^. Cases that did not meet these criteria or individuals who did not show any symptoms were classified as asymptomatic. All univariate and multivariate analyses were performed using Minitab software, version 14.0. Graphs were generated in GraphPad version 7.0 and Excel 2010.

### Ethics approval and consent to participate

The study was submitted to and approved by the National Research Ethics Committee (CONEP, acronym in Portuguese) and the Human Research Ethics Committee of the Health Sciences Institute of Federal University of Pará (UFPA) under CAAE n. 31800720.1.0000.0018 in compliance with the guidelines and regulatory standards for research involving human beings, with all methods conducted in accordance with the Declaration of Helsinki.

## Results

Socioeconomic and behavioral characteristics and comorbidities associated with the risk of SARS-CoV-2 infection.

Between October 2020 and February 2021, 736 individuals were invited and agreed to participate in the study. Based on serological results, 275 (37.3%) individuals were reactive for anti-SARS-CoV-2 IgG antibodies, 429 (58.2%) were nonreactive, and 32 (4.3%) had indeterminate results. In total, only 704 (100%) individuals with a confirmed laboratory diagnosis were eligible for statistical analysis; those with indeterminate results were excluded from the analysis. The prevalence of anti-SARS-CoV-2 IgG antibodies was higher in women (F = 65.1%/M = 34.9%; p = 0.4445) than in men. Advanced age ≥ 70 years (odds ratio (OR) = 2.02; 95% CI 1.02–3.05; p = 0.044), a high school education level (OR = 2.41; 95% CI 1.08–2.16; p = 0.016), mixed race (self-report; OR = 3.24; 95% CI 1.23–2.35; p = 0.001) and income ≤ 2 minimum wages (OR = 2.39; 95% CI 1.08–2.12; p = 0.017) were associated with a higher risk of infection (Table 1). The most frequent comorbidities were hypertension (24.7%) and asthmtic (9.1%). No correlation was found between seropositivity for anti-SARS-CoV-2 antibodies and the presence of comorbidities (Table 1).

**Table 1:** Prevalence of socioeconomic characteristics and comorbidities and the risk of SARS-CoV-2 infection.

Variables	Reagents (%)	No reagents (%)	Univariate analysis OR (95% CI)	*p*	Multivariate analysis OR (95% CI)	*p*
**Total**	275 (39.1)	429 (60.9)				
**Age**						
≤ 18	18 (26.9)	49 (11.4)	(1 Ref)			
19–29	55 (47.4)	75 (17.5)	0.79 (0.79–1.76)	0.429		
30–39	29 (31.9)	62 (14.4)	1.48 (0.44–1.12)	0.138		
40–49	48 (38.4)	77 (17.9)	0.14 (0.65–1.45)	0.886		
50–59	55 (44.7)	69 (16.1)	1.44 (0.90 -1.98)	0.151		
60–69	37 (37.0)	63 (14.7)	0.44 (0.59–1.41)	0.663		
≥ 70	30 (47.6)	33 (7.7)	1.47 (0.88 -2.48)	0.142	2.02 (1.02–3.05)	**0.044**
NI	3 (15.8)	1 (0.2)				
**Education**						
Elementary school	38 (13.8)	66 (15.4)	0.58 (0.57–1.35)	0.559		
High school	110 (40.0)	125 (29.1)	2.95 (1.17 -2.22)	**0.003**	2.41 (1.08–2.16)	**0.016**
Graduate	89 (32.4)	157 (36.6)	1.17 (0.60–1.14)	0.242		
Undergraduate	38 (13.8)	80 (18.6)	(1 Ref)			
NI	0	1 (0.2)				
**Race**						
Yellow	3 (1.1)	9 (2.1)	0.99 (0.14–1.92)	0.322		
White	62 (22.5)	146 (34.0)	3.31 (0.39–0.79)	**0.001**		
Black	36 (13.1)	56 (13.1)	(1 Ref)			
Mixed	173 (62.0)	213 (49.7)	3.34 (1.24–2.31)	**0.001**	3.24 (1.23–2.35)	**0.001**
NI	1 (0.4)	5 (1.2)				
**Income**						
≤ 1–2	135 (41.9)	159 (37.1)	3.15 (1.20–2.23)	**0.002**	2.39 (1.08–2.12)	**0.017**
3–4	53 (24.7)	127 (29.7)	3.15 (0.45 -0.83)	**0.002**		
≥ 5	79 (23.3)	127 (29.7)	(1 Ref)			
NI	8 (2.9)	16 (3.7)				
**Comorbidity**						
Diabetes	22 (8.0)	34 (7.9)	1.02 (0.58 -1.78)	0.957	*	*
Asthma	25 (9.1)	39 (9.1)	0.93 (0.5–1.59)	0.803	*	*
Autoimmune disease	5 (1.8)	10 (2.3)	1.38 (0.50–3.85)	0.537	*	*
Cardiovascular disease	11 (4.0)	17 (4.0)	0.58 (0.26–1.34)	0.205	*	*
Hypertension	68 (24.7)	96 (22.4)	1.25 (0.88–1.78)	0.220	*	*
Obesity	13 (4.7)	26 (6.1)	1.07 (0.55–2.11)	0.839	*	*
Tuberculosis	1 (0.4)	3 (0.7)	4.74 (0.49–4.74)	0.179	*	*
None	157 (57.1)	250 (58.3)	(1 Ref)			

*OR* odds ratio, *NI* not informed.

Significant values are in bold.

*Not significant on multivariate analysis.

**Figure 1:**
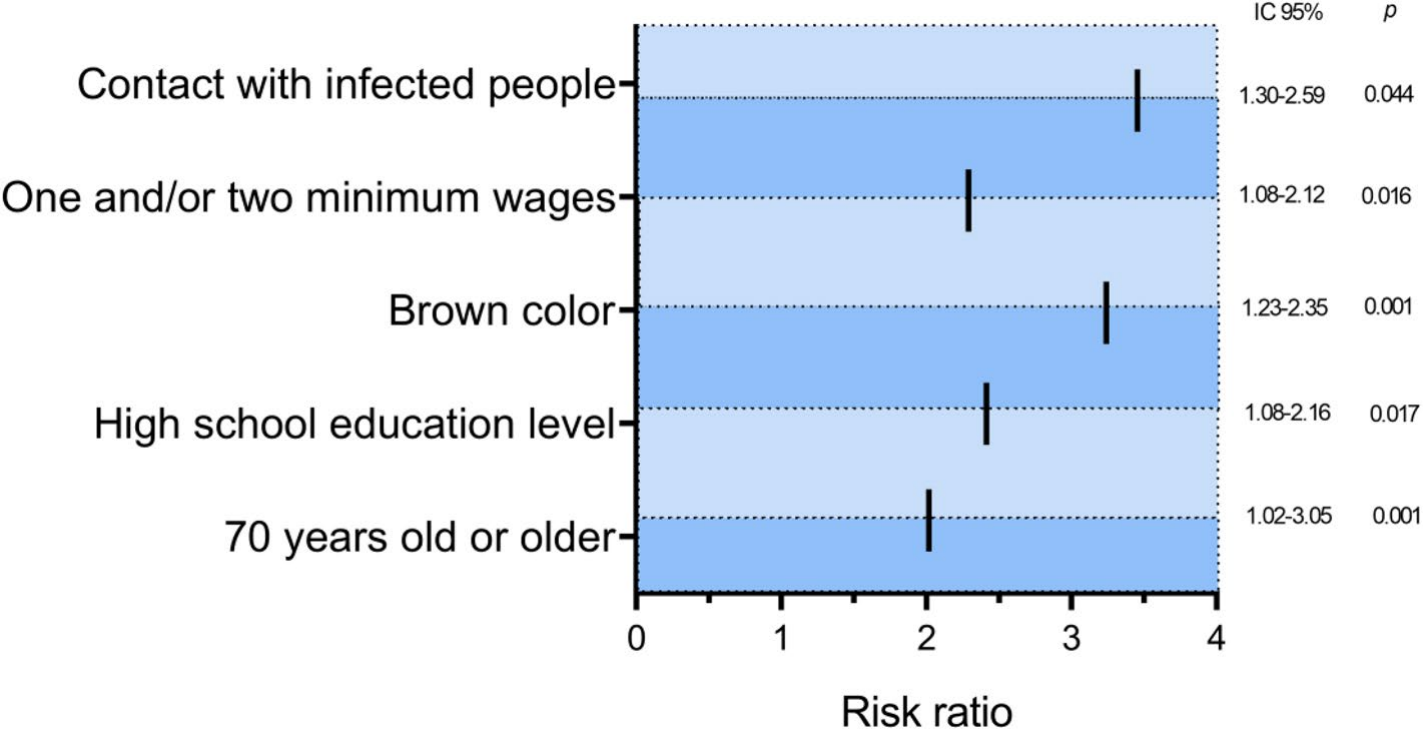
Main risk factors for SARS-CoV-2 infection observed in the study population.

Behavioral patterns considered important for prevention, such as mask use, hand washing and lack of travel during the pandemic, were not associated with the risk of SARS-CoV-2 infection (p > 0.005) (Table 2). Interestingly, the prevalence of antibodies was higher among individuals who had been under lockdown (85.1%; OR = 2.11; 95% CI 1.04–2.67; p = 0.035), which was not considered a risk factor for infection. However, contact with infected individuals (OR = 3.45; 95% CI 1.30–2.59; p = 0.001) may be a risk factor for SARS-CoV-2 infection, in addition to other socioeconomic factors (Fig. 1).

**Table 2:** Seroprevalence of behavioral characteristics associated with SARS-CoV-2 infection.

Variables	Reagents (%)	No reagents (%)	Univariate analysis OR (95% CI)	p	Multivariate analysis OR (95% CI)	p
Total	275 (39.1)	429 (60.9)				
**Contact with an infected person**						
Yes	82 (29.8)	37 (8.6)	2.68 (1.13–2.15)	**0.007**	3.45 (1.30–2.59)	**0.001**
No	189 (68.7)	380 (88.6)	(1 Ref)			
NI	4 (1.5)	12 (2.8)				
**Social isolation**						
Yes	234 (85.1)	389 (90.7)	(1 Ref)			
No	39 (14.2)	39 (9.1)	2.11 (1.04–2.67)	**0.035**	*	*
NI	2 9 (0.7)	1 (0.2)				
**Handwashing**						
Rarely	24 (8.7)	171 (39.9)	0.05 (0.58–1.69)	0.959	*	*
Many times a day	250 (90.9)	253 (59.0)	(1 Ref)			
NI	1 (0.4)	5 (1.1)				
**Mask use**						
Sometimes	18 (6.5)	20 (4.6)	− 1.06 (0.70–0.36)	0.290	*	*
Always	245 (89.1)	388 (90.4)	(1 Ref)			
NI	12 (4.4)	21 (4.9)				
**Travel**						
Yes	106 (38.5)	182 (42.4)	0.85 (0.63–1.16)	0.320	*	*
No	167 (60.7)	245 (51.7)	(1 Ref)			
NI	2 (0.8)	2 (0.5)				

*OR* odds ratio, *NI* not informed.

Significant values are in bold.

*Not significant on multivariate analysis.

### Antibody seroprevalence among symptomatic and asymptomatic individuals

**Figure 2:**
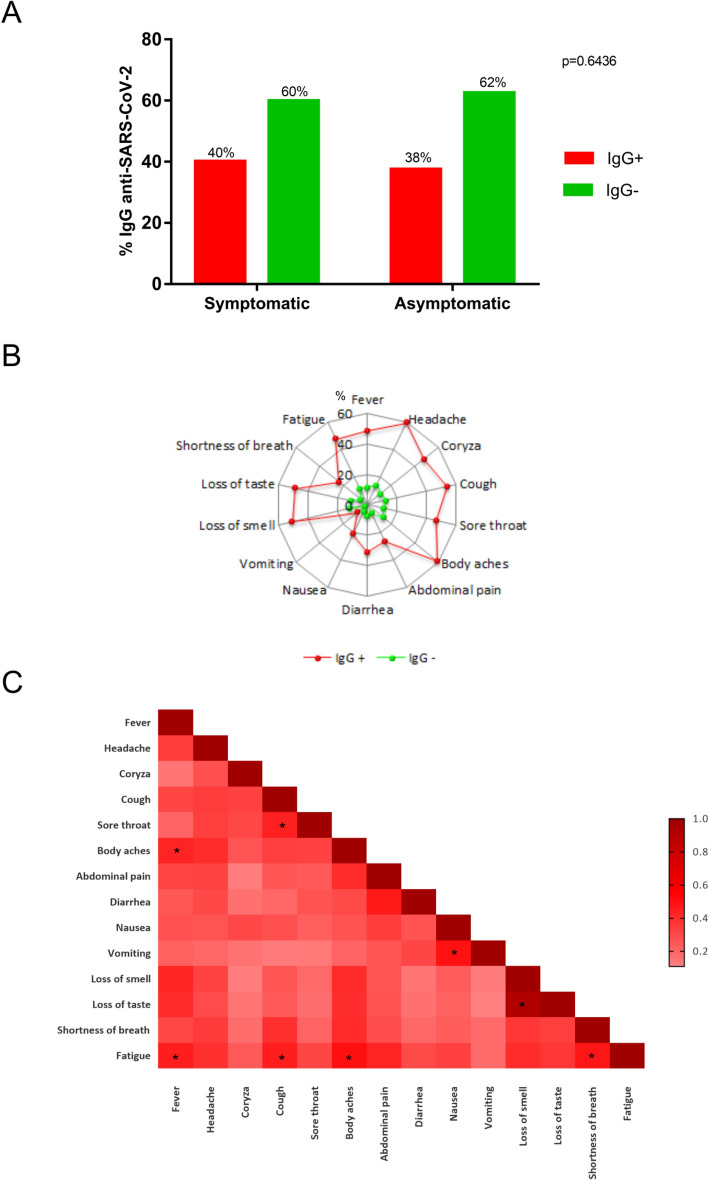
Prevalence of antibodies and symptoms among symptomatic and asymptomatic individuals. (**A**) Percentage of  ± anti-SARS-CoV-2 IgG antibodies between symptomatic and asymptomatic individuals. (**B**) Prevalence of symptoms characteristic of COVID-19. (**C**) Frequency of the cooccurrence of symptom pairs in symptomatic individuals. Values with * correspond to p values < 0.05.

Approximately 62.5% of the individuals were classified as symptomatic, and 37.5% were classified as asymptomatic. No significant difference in seropositivity was noted between the groups (Fig. 2A). The presence of characteristic COVID-19 symptoms was higher in IgG + individuals. Symptoms such as fever (48.7%; p = 0.0001), cough (53.3%; p = 0.0206), body aches (58.9%; p = 0.0001), abdominal pain (26.5%; p = 0.0203), loss of smell (50.5%; p = 0.0001), loss of taste (48.4%; p = 0.0001), shortness of breath (24.0%; p = 0.0007) and fatigue (48.0%; p = 0.0001) were more prevalent among seropositive individuals (Fig. 2B) (Table 3). Among the symptoms, the correlation between loss of smell and taste was highly significant in symptomatic patients (Fig. 2C). Among the variables analyzed, only individuals in the age groups of 40–49 years (OR = 1.99; 95% CI 1.01–3.75; p = 0.047) and 50–59 years (OR = 2.62; 95% CI 1.28–5.62; p = 0.009) were more susceptible to the presence of clinical symptoms related to COVID-19. No other variable was correlated with the presence of symptoms (p > 0.005).

**Table 3:** Prevalence of symptoms characteristic of COVID-19 in IgG + individuals.

Symptoms	Reagents (%)	No reagents (%)	*p*
Total	275 (39.1)	429 (60.9)	
Fever	134 (48.7)	116 (27.0)	** < 0.0001**
Headache	165 (60.0)	234 (54.5)	0.1542
Coryza	131 (47.6)	210 (49.0)	0.7334
Cough	147 (53.5)	191 (44.5)	**0.0206**
Sore throat	127 (46.2)	193 (45.0)	0.7563
Body aches	162 (58.9)	165 (38.5)	** < 0.0001**
Abdominal pain	73 (26.5)	82 (19.1)	**0.0203**
Diarrhea	86 (31.3)	129 (30.1)	0.7353
Nausea	59 (21.5)	88 (20.5)	0.7642
Vomiting	22 (8.0)	50 (11.7)	0.1184
Loss of smell	139 (50.5)	61 (14.2)	** < 0.0001**
Loss of taste	133 (48.4)	68 (15.9)	** < 0.0001**
Shortness of breath	66 (24.0)	60 (14.0)	**0.0007**
Fatigue	132 (48.0)	143 (33.3)	** < 0.0001**

Significant values are in bold.

## Discussion

We estimated the prevalence of anti-SARS-CoV-2 IgG antibodies for the S1 subunit of the spike glycoprotein and factors potentially associated with SARS-CoV-2 infection 6 months after the beginning of the first wave of COVID-19 in Belém, Pará, Brazil. To our knowledge, this is one of the first seroepidemiological studies describing the prevalence of anti-SARS-CoV-2 antibodies in the city of Belém.

The seroprevalence of anti-SARS-CoV-2 antibodies varies based on geographic region, study population, sampling time, laboratory method and COVID-19 incidence at the sampling site. In Brazil, the prevalence of antibodies was highly heterogeneous when considering the five regions of the country, with the North and Northeast regions clearly having the highest seroprevalence rates, which were strongly associated with low socioeconomic status^12,13^. Two of the highest seroprevalence estimates in Brazil were reported in Manaus at 29.10% and in the capital of Maranhão, with an antibody prevalence of approximately 40.4% in the population^14–16^. Our results indicate a seropositivity of 39.24% in the city of Belém. We believe that the seroprevalence in Belém may be even higher than that observed when we consider our previous results of a follow-up of a post-COVID-19 cohort in which we found a 30% loss of anti-SARS-CoV-2 IgG antibodies 3 months after COVID-19 diagnosis^17^. The choice of serological test was a determining factor for the prevalence rate of antibodies found in this study because due to good sensitivity and specificity, the test allowed the detection of asymptomatic seropositive individuals who usually do not seek medical care or undergo diagnostic tests and are therefore underreported in official numbers. According to the Brazilian Institute of Geography and Statistics, the population of the capital city of Belém is 1.5 million inhabitants^18^. Based on our seroprevalence results, which indicated that 39% of participants had been exposed to SARS-CoV-2, the estimated number of infected individuals in the capital would be 585,000, which is 10 times higher than the number reported by the Municipal Department of Health as of December 30, 2020^19^, as these reports are based only on cases of symptomatic individuals seeking diagnosis and treatment services. We believe that the seroprevalence results observed in the present study are not influenced by possible false-positive or false-negative results, since a methodology with high sensitivity and specificity was used.

Our study failed to identify a significant difference between the sexes regarding the risk of SARS-CoV-2 infection, which is similar to findings reported in other seroprevalence studies^20,21^. The presence of comorbidities was not a predictor of positivity for anti-SARS-CoV-2 IgG antibodies, which is similar to observations in another study^22^. However, comorbidities may be associated with a high risk of serious complications and death due to COVID-19^23,24^. The age group most exposed to the virus and with the highest seroprevalence rates was adults aged over 70 years, which is similar to observations in other studies^25,26^. As home lockdown was voluntary, the recommendations for social lockdown may not have been adequately followed, suggesting exposure at the family level. The antibody rate by age can be a useful tool to track vaccine prioritization for the population, as defined by the National Plan for the Operationalization of Vaccination against COVID-19 of the Brazilian Ministry of Health^27^.

Individuals who self-reported being mixed race have a high prevalence of antibodies and a high risk of viral infection because they represent the majority of the Brazilian population. Our results demonstrate a robust association between socioeconomic conditions and the risk of SARS-CoV-2 infection, with a higher risk for individuals with a high school education and with a family income less than or equal to one or two minimum wages—socioeconomic characteristics that are prevalent in low- and middle-income countries such as Brazil. Family income was the most significant risk factor. Families with incomes between three and five times the minimum wage were less likely to be infected than were families with lower incomes, which is similar to the rest of the country^12,13,28^. Socioeconomic inequalities became even more evident with the pandemic. We assume that social health differences contributed to these initial observations and resulted in differential exposure to the virus and differential vulnerability to infection, indicating the need for preparedness plans for vulnerable communities to ensure a rapid and coordinated response to protect these groups from future pandemics or crises at an early stage.

Regarding infection prevention measures, interestingly, we found that individuals who regularly used a mask and frequently engaged in hand hygiene had the highest seroprevalence rates. Notably, our data suggest that the lack of information on proper handwashing techniques, proper mask use and mask efficiency explain the high prevalence of IgG anti-SARS-CoV-2 antibodies in this population. Our data indicate that direct contact with individuals infected with SARS-CoV-2 increases the risk of infection, which is consistent with results reported in other studies^29,30^.

Our results indicate a relatively high seroprevalence of asymptomatic SARS-CoV-2 infection (37.5%) in the city of Belém, corroborating other studies conducted in Brazil^16,31^, which complicates disruption of the transmission cycle, since a high percentage of individuals who claimed to have had no symptoms of COVID-19 were seropositive, thus highlighting the possibility of silent transmission of the virus by asymptomatic individuals.

Mass testing is important for rapid identification of asymptomatic cases and isolation of infected individuals, with testing and monitoring of their contacts, to prevent the spread of the virus^32^. Clinical symptoms such as fever, body aches, loss of smell, loss of taste, shortness of breath and fatigue are strongly associated with SARS-CoV-2 infection, with studies showing symptom profiles similar to those found in our population. In March 2020, the first reports relating the loss of smell and/or taste with COVID-19 were published^33,34^. The available data suggest a prevalence of loss of smell and/or taste ranging from 31 to 85% among patients with COVID-19^33–37^. Our results support that otorhinolaryngological symptoms are prevalent among patients with mild to moderate COVID-19 infection because we observed a high prevalence of these symptoms in participants with mild or moderate infection and positive results for anti-SARS-CoV-2 IgG antibodies.

In Belém, we observed that individuals aged 40–59 years had a higher risk of developing symptoms than younger people. A retrospective cohort study found that most hospitalized patients with COVID-19 symptoms had a mean age of 54 years and that advanced age was a risk factor for death among adults^38^. The effects of age on immune system function, the activity of T and B lymphocytes and excess production of pro-inflammatory cytokines influence the onset of symptoms owing to poor control of viral replication^39^. Thus, the correlation between age and the risk of SARS-CoV-2 infection or the risk of developing critical illness observed in this study may be essential information for planning health resources to ensure the sustainability of the system.

All countries are urged to make increasingly rapid decisions regarding socioeconomic issues and public health policies to contain the pandemic. High-quality data, as presented here, are necessary to facilitate decision making. This study provides important information about the high prevalence of SARS-CoV-2 in the city of Belém and indicates that individuals with low socioeconomic status and/or little education are more susceptible to infection—a pattern commonly found in low-income countries. We also discovered the impact of the proper use of prevention and control measures and suggest that preventive measures are essential to minimize symptomatic and asymptomatic transmission in the community. Seroprevalence surveys will be needed to identify dynamic trends and monitor disparities to profile future transmission dynamics and formulate protective responses for vulnerable groups.

We understand that the present study has some limitations. We encountered difficulties when obtaining samples due to the lockdown and social distancing decrees related to the pandemic, which may have led to a sample bias in the statistical analysis. Nevertheless, our analyses have strengths. The study population was representative of both sexes, all ages and different economic groups and races. The serological test based on the S protein can detect past infections, including asymptomatic and recovered cases, providing better information on the prevalence of the disease in the population. To date, this is one of the first epidemiological studies to investigate risk factors for SARS-CoV-2 infection in the city of Belém, northern Brazil.

All countries are urged to make increasingly rapid decisions regarding socioeconomic issues and public health policies to contain the epidemic. We were able to estimate the high seroprevalence of SARS-CoV-2 in the first six months after the first wave of COVID-19 in the city of Belém and identify the factors that may be driving seropositivity and exposure to SARS-CoV-2 in this population. These findings highlight the importance of serosurveillance for estimating the real impact of the COVID-19 pandemic, identifying trends in viral transmission and monitoring population disparities to profile future transmission dynamics and formulate protective responses for vulnerable groups. In addition, these results may be essential for monitoring the evolution of the pandemic after easing social distancing measures and the implementation of a vaccination schedule in Brazil as a basis for other countries.

## Data Availability

All data supporting the findings of this study are available in the article.
